# Association of varicose veins with the risk of heart failure: A nationwide cohort study

**DOI:** 10.1371/journal.pone.0316942

**Published:** 2025-01-07

**Authors:** Dongyeop Kim, Moo-Seok Park, Ju-young Park, Tae-Jin Song

**Affiliations:** 1 Department of Neurology, Seoul Hospital, Ewha Womans University College of Medicine, Seoul, South Korea; 2 Busan Center for Medical Mathematics, National Institute for Mathematical Sciences, Seo-gu, Busan, Republic of Korea; Kyung Hee University School of Medicine, REPUBLIC OF KOREA

## Abstract

**Background:**

Research investigating the association between varicose veins (VV) and heart failure has been limited. Here, we examine this association within a nationwide longitudinal cohort, hypothesizing an increased risk of heart failure associated with the presence of VV.

**Methods:**

Our study included 390,436 participants based on health screening results conducted from 2005 to 2010 in the South Korean health screening cohort database. Presence of VV was defined as having at least two claims based on International Classification of Diseases, Tenth Revision (ICD-10) codes I830-832, I839, or I868. Propensity score matching (PSM) at a ratio of 1:5 was employed to categorize the participants into two groups based on the presence of VV. The primary outcome, heart failure incidence, was defined as two or more claims with ICD-10 code I50 during follow-up.

**Results:**

Among the participants, presence of VV was noted in 5,008 (1.28%) individuals. Over a median follow-up period of 13.33 years (interquartile range 10.4–16.26), 55,023 cases of heart failure (14.0%) occurred. In the multivariable analysis, the group with VV consistently showed an increased incidence risk of heart failure compared to the group without VV, both before (hazard ratios [HR], 1.174; 95% confidence interval [CI], 1.089–1.265) and after PSM (HR, 1.171; 95% CI, 1.070–1.283). Landmark analysis also found a consistent relationship between the presence of VV and the incidence risk of heart failure before (HR, 1.190; 95% CI, 1.103–1.285) and after PSM (HR, 1.144; 95% CI, 1.044–1.254).

**Conclusions:**

This study revealed a significant increase in the risk of heart failure among patients with VV in the general population of South Korea. Given that the presence of VV is likely associated with an increased risk of heart failure in the general population, the potential for future heart failure should be taken into consideration when VV are present.

## Introduction

Varicose veins (VV) are superficial veins that are enlarged and twisted, typically with a diameter of three millimeters or more in the lower limbs. They are classified as a sub-category of chronic venous disease, corresponding to C2 in the Clinical-Etiological-Anatomical-Pathophysiological (CEAP) classification system [1]. These veins, located beneath the skin, primarily affect the greater and lesser saphenous veins and their branches, as well as other superficial veins in the legs that are not part of the saphenous system [2]. The risk of developing VV increases due to various factors including old age, female sex, prolonged standing, sedentary activities, obesity, pregnancy, smoking, drinking, and family history [3].

Heart failure is a medical condition in which the heart fails to adequately supply the body with the blood it requires due to dysfunction in either pumping blood out of the heart or filling with blood [4]. It is a prevalent condition globally, with increasing incidence. Despite advancements in treatment and preventive measures, rates of illness and mortality associated with heart failure remain substantial [5]. Therefore, it is crucial to recognize the risk factors for heart failure. Established risk factors include older age, male sex, hypertension, left ventricular hypertrophy, myocardial infarction, valvular heart disease, obesity, diabetes, and various other clinical conditions [6].

Both VV and heart failure are prevalent diseases worldwide, significantly impacting the quality of life [5, 7, 8]. Recent reports indicate that chronic venous disease may be linked to an increased risk of arterial diseases, heart failure, and long-term mortality [9, 10]. However, there has been limited research on the connection between the incidence risk of heart failure and chronic venous disease. We hypothesized that the presence of VV would be associated with an increased risk of heart failure in a longitudinal study setting. In this study, we investigated the association between the presence of VV, as well as its treatment, and the incidence risk of heart failure in a nationwide longitudinal study of the general population.

## Methods

### Data source

This study utilized the National Health Insurance Service-Health Screening (NHIS-HEALS) cohort database in South Korea. The NHIS-HEALS database encompasses a nationwide free health screening program conducted biennially for all South Korean adults over the age of 40 [11]. The NHIS-HEALS cohort was established by randomly selecting 10% of participants who underwent health examinations in 2002 and 2003. Our study incorporated health examination data from this cohort, covering the period from 2002 to 2019. The health screening program includes a wide range of health assessments, including blood pressure, body mass index (BMI), blood chemistry profiles, and a self-administered questionnaire on medical history, as well as lifestyle habits such as physical activity, smoking, and alcohol consumption. Demographic data including sex, age, and household income are also included. Moreover, the dataset encompasses comprehensive health claim data for all participants, including hospital visits, diagnoses, surgeries, medical procedures, and prescriptions. Hospital visits and diagnoses were classified according to the International Classification of Diseases, Tenth Revision (ICD-10). Information on health claims, insurance coverage, and participant mortality status was available until December 31, 2019. Due to the retrospective nature of the NHIS data analysis, which does not include personally identifiable information, informed consent was waived for this study. This research was approved by the Institutional Review Board of Ewha Womans University Seoul Hospital (SEUMC 2024-03-006).

### Study population

**Fig 1:**
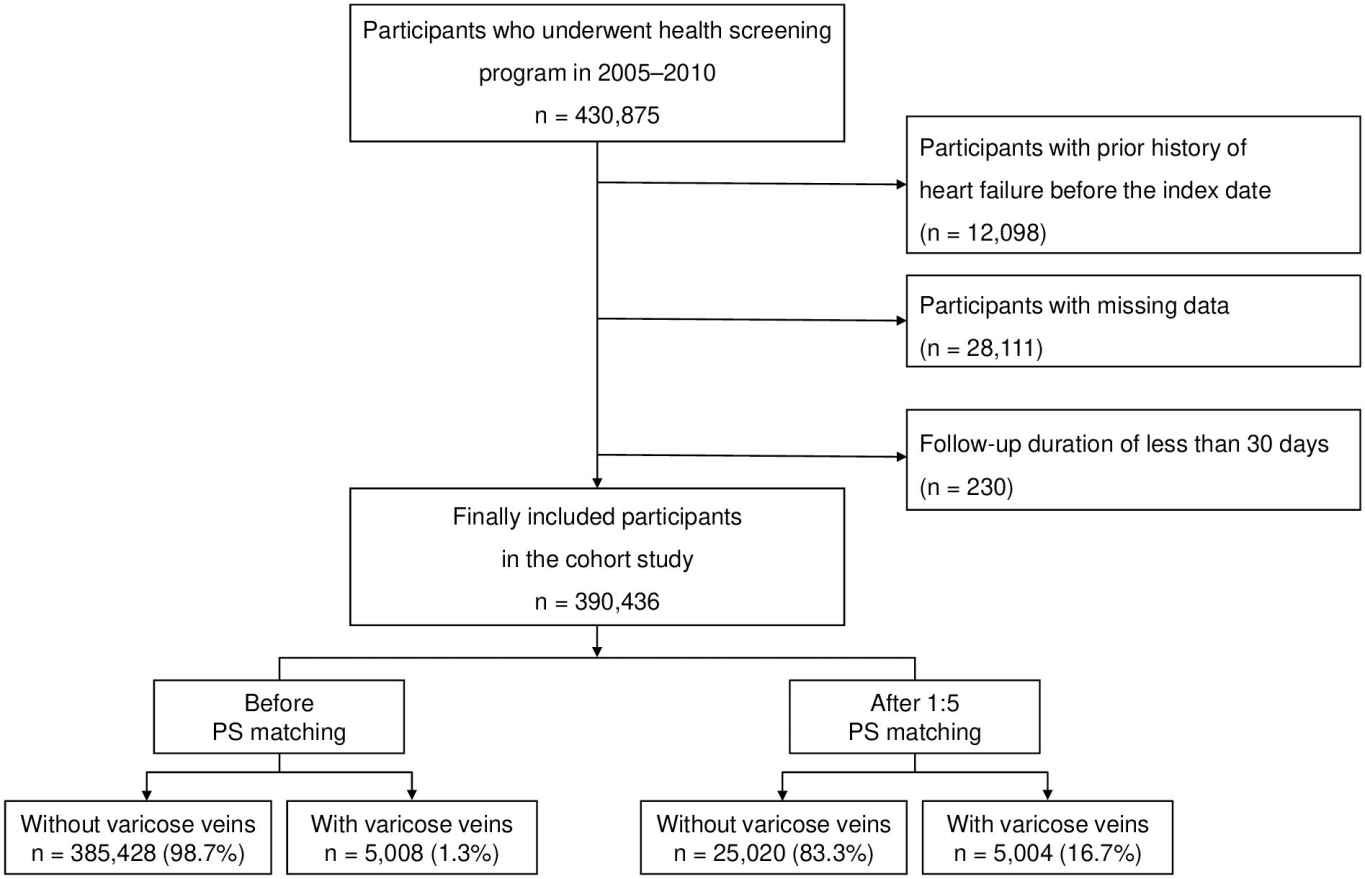
Flow chart of inclusion and exclusion.

To ensure an adequate number of VV cases and a sufficient follow-up period, the study enrolled 430,875 participants from the NHIS-HEALS cohort database who underwent the national health screening program between 2005 and 2010. Follow-up data were collected until December 31, 2019. To ensure the exclusion of subjects with a history of heart failure, a washout period was defined from January 1, 2002, to the baseline (2005–2010). During this period, patients diagnosed with heart failure under the ICD-10 code I50 were excluded (n = 12,098) [12, 13]. Furthermore, participants with missing data (n = 28,111) and those with less than 30 days of follow-up (n = 230) were also excluded. Finally, 390,436 participants were included (Fig 1).

### Definition of varicose veins, heart failure, and covariates

Based on health claims data in NHIS-HEALS, the VV group was defined as individuals diagnosed with VV two or more times during the follow-up period, identified by the ICD-10 diagnostic codes: I830–832, I839, or I868, which were previously validated [14]. In addition, the treatment or procedure code for VV was defined according to ICD-10 diagnostic codes O0261–267 (open surgery including stripping, saphenous vein ligation, or phlebectomy), O2052 (local resection), and O0215–217 (sclerotherapy). The primary outcome was the incidence of heart failure, defined as two or more claims based on ICD-10 code I50 during the follow-up period [15]. To elucidate potential covariates influencing heart failure onset, we collated demographic and clinical variables including sex, age, BMI, socioeconomic status determined by income level, smoking history, frequency of alcohol consumption, and a comprehensive profile of comorbid conditions along with the Charlson comorbidity index (CCI) at the index date [16]. Income level was determined using percentiles of individual health insurance premiums; smoking status was categorized as past smoking history, current smokers, and non-smokers; and alcohol intake frequency was defined as the number of times alcohol was consumed per week. Detailed definitions of comorbidities are provided in S1 Appendix [17–20].

### Statistical analysis

Comparisons between the VV group and non-VV group were conducted using an independent t-test for continuous variables and a Chi-square test (or Fisher’s exact test) for categorical variables [21]. To balance baseline characteristics and control for potential confounders between these two groups, we employed propensity score matching (PSM) [22, 23]. In this study, 1:5 PSM was conducted between the VV group and non-VV group, with propensity scores calculated based on sex, age, BMI, income level, smoking status, frequency of alcohol intake, comorbidities, and the CCI. The adequacy of PSM was assessed using standardized mean differences (SMDs), and PSM was considered appropriate when the absolute values of SMDs were less than 0.1 [24, 25]. To analyze the risk of outcomes, we used Kaplan-Meier survival curves and tested the differences between groups with log-rank tests. In addition, Cox proportional hazard models were used to calculate hazard ratios (HR) and 95% confidence intervals (CI). In analyzing PSM data, we applied stratified proportional hazard models to improve accuracy by considering paired matching data characteristics. The proportional hazards assumption for covariates in Cox proportional hazard models was assessed using Grambsch and Therneau’s test of scaled Schoenfeld residuals, yielding satisfactory results. Multivariable Cox proportional hazard models and stratified proportional hazard were adjusted for sex, age, BMI, income level, smoking status, alcohol consumption frequency, comorbidities, and CCI. Furthermore, sensitivity analyses were performed to assess the risk ratios of VV in sub-groups of each clinical variable using Cox proportional hazard models and stratified proportional hazard, and these were visualized using forest plots. To minimize reserve causality, landmark analysis was performed with the outcome being the case where heart failure occurred 1 year after the index date. A Fine and Gray competing risk regression model was incorporated to account for the competing risk of mortality in the analysis of heart failure risk associated with VV, with subhazard ratios (SHR) and 95% CI calculated to provide a more accurate estimation of heart failure risk. All statistical analyses were performed using SAS 9.4 version (SAS Inc., Cary, NC, USA) and R software, version 4.2.1 (R Foundation for Statistical Computing, Vienna, Austria). We considered statistical significance for all our tests as a two-sided *P*-value less than 0.05.

## Results

### Baseline characteristics of participants

Baseline characteristics according to the presence of VV are provided in Table 1. The mean age of the participants was 56.0 ± 9.3 years, and 54.3% were male. The presence of VV was noted in 5,008 (1.28%) participants, who were predominantly female. Those with VV were less likely to be current smokers, heavy alcohol users, or diabetes mellitus patients. However, they were more likely to have comorbidities such as dyslipidemia, chronic obstructive pulmonary disease, renal disease, liver disease, and cancer compared to those without VV. After PSM, the populations with and without VV were well-balanced in terms of matching.

**Table 1:** Baseline characteristics of study participants.

Variables	Before PSM				After PSM 1:5		
	Total	Varicose veins (-)	Varicose veins (+)	p-value	Varicose veins (-)	Varicose veins (+)	SMD*
		Mean ± SD,	Mean ± SD,		Mean ± SD,	Mean ± SD,	
		n (%)	n (%)		n (%)	n (%)	
Number	390,436	385,428	5,008		25,020	5,004	
Age, years	56.0 ± 9.3	56.0 ± 9.3	56.8 ± 8.2	< .001	56.6 ± 9.3	56.8 ± 8.2	-0.023
Sex				< .001			-0.015
Female	178,487 (45.7)	175,631 (45.6)	2,856 (57.0)		14,458 (57.8)	2,855 (57.0)	
Male	211,949 (54.3)	209,797 (54.4)	2,152 (43.0)		10,562 (42.2)	2,149 (43.0)	
Body mass index (kg/m^2^)	24.0 ± 2.9	24.0 ± 2.9	24.1 ± 2.8	< .001	24.1 ± 2.9	24.1 ± 2.8	0.001
Household income				.002			0.000
Q1	112,719 (28.9)	111,386 (28.9)	1,333 (26.6)		6,665 (26.6)	1,333 (26.6)	
Q2	141,751 (36.3)	139,874 (36.3)	1,877 (37.5)		9,367 (37.4)	1,874 (37.5)	
Q3	135,966 (34.8)	134,168 (34.8)	1,798 (35.9)		8,988 (36.0)	1,797 (35.9)	
Smoking status				< .001			0.007
Never	278,197 (71.3)	274,270 (71.2)	3,927 (78.4)		19,687 (78.7)	3,924 (78.4)	
Former	36,692 (9.4)	36,160 (9.4)	532 (10.6)		2,662 (10.6)	532 (10.6)	
Current	75,547 (19.3)	74,998 (19.4)	549 (11.0)		2,671 (10.7)	548 (11.0)	
Alcohol consumption (days/week)				< .001			0.001
None	234,576 (60.1)	231,316 (60.0)	3,260 (65.1)		16,309 (65.2)	3,259 (65.1)	
1–2 times	115,278 (29.5)	113,971 (29.6)	1,307 (26.1)		6,535 (26.1)	1,306 (26.1)	
3–4 times	25,400 (6.5)	25,109 (6.5)	291 (5.8)		1,431 (5.7)	290 (5.8)	
≥ 5 times	15,182 (3.9)	15,032 (3.9)	150 (3.0)		745 (3.0)	149 (3.0)	
Regular physical activity (days/week)				< .001			0.001
None	190,726 (48.9)	188,773 (50.0)	1,953 (39.0)		9,772 (39.1)	1,953 (39.0)	
1–4 days	151,927 (38.9)	149,907 (38.9)	2,020 (40.3)		10,275 (41.1)	2,019 (40.4)	
≥ 5 days	47,783 (12.2)	46,748 (12.1)	1,035 (20.7)		4,973 (19.8)	1,032 (20.6)	
Comorbidities							
Hypertension	94,224 (24.1)	93,021 (24.1)	1,203 (24.0)	.853	5,889 (23.5)	1,203 (24.0)	-0.012
Diabetes mellitus	41,531 (10.6)	41,090 (10.7)	441 (8.8)	< .001	2,134 (8.5)	441 (8.8)	-0.010
Dyslipidemia	104,591 (26.8)	102,626 (26.6)	1,965 (39.2)	< .001	9,776 (39.1)	1,965 (39.3)	-0.004
Stroke	2,232 (0.6)	2,197 (0.6)	35 (0.7)	.229	174 (0.7)	35 (0.7)	0.000
Myocardial Infarction	770 (0.2)	761 (0.2)	9 (0.2)	.779	43 (0.2)	9 (0.2)	-0.002
COPD	118,235 (30.3)	116,160 (30.1)	2,075 (41.4)	< .001	10,336 (41.3)	2,075 (41.5)	-0.003
Renal disease	14,351 (3.7)	14,095 (3.7)	256 (5.1)	< .001	1,243 (5.0)	256 (5.1)	-0.007
Liver disease	89,685 (23.0)	88,037 (22.8)	1,648 (32.9)	< .001	8,216 (33.8)	1,648 (32.9)	-0.002
Cancer	21,715 (5.6)	21,335 (5.5)	380 (7.6)	< .001	1,812 (7.2)	380 (7.6)	-0.014
Charlson comorbidity index				.526			-0.001
0	360,948 (93.5)	356,249 (93.5)	4,699 (93.9)		23,487 (93.9)	4,699 (93.9)	
1	22,802 (5.9)	22,525 (5.9)	277 (5.5)		1,402 (5.6)	277 (5.5)	
≥ 2	2,209 (0.6)	2,181 (0.6)	28 (0.6)		131 (0.5)	28 (0.6)	

Abbreviations: PSM, propensity score matching; SD, standard deviation; Q, quantile; COPD, chronic obstructive pulmonary disease; SMD, standardized mean difference.

*All standardized mean difference values were < 0.1 in the propensity score matched cohort.

### Association of presence of varicose veins with heart failure

**Fig 2:**
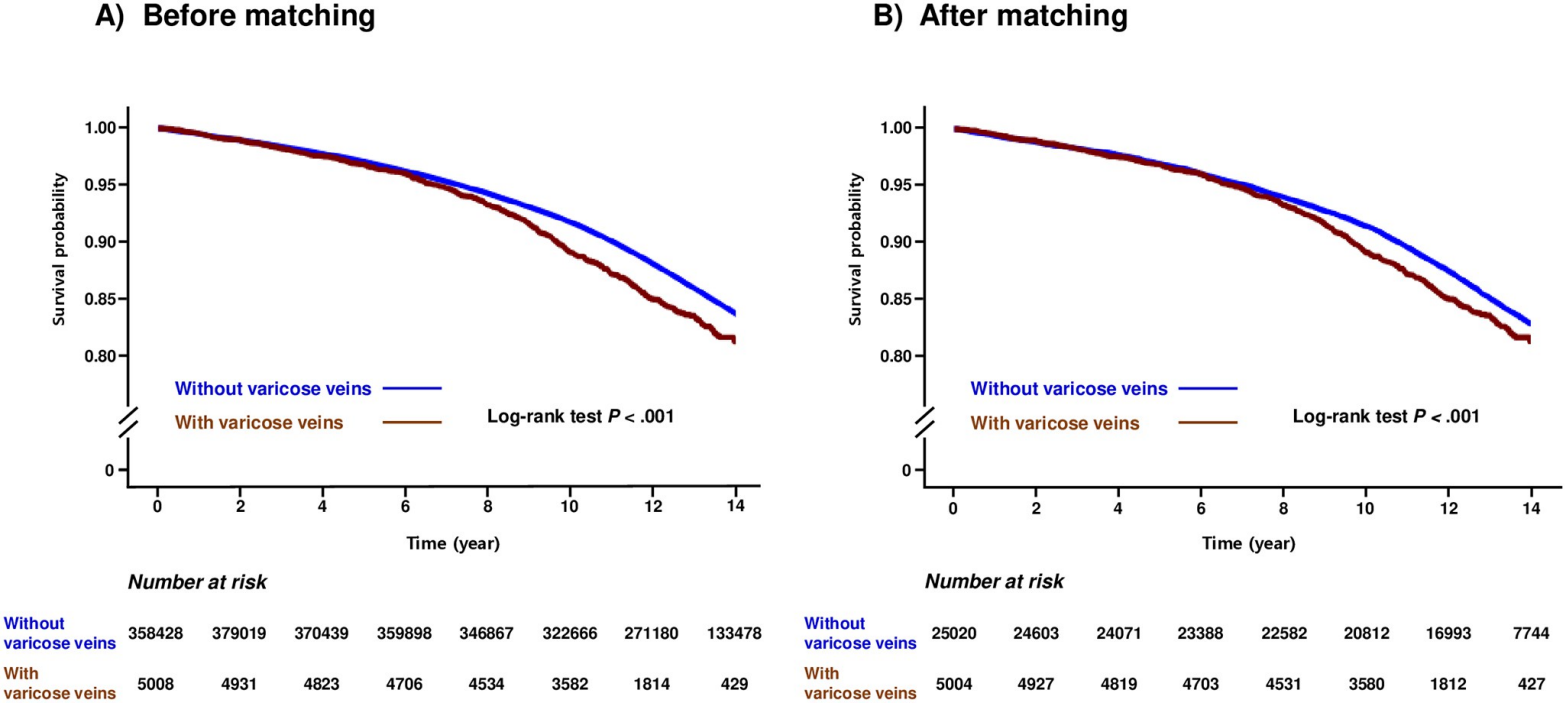
Kaplan-Meier survival curves illustrating the association between heart failure outcome and the presence of varicose veins.

During a median follow-up of 13.33 years (interquartile range, 10.4–16.26), 55,023 cases of heart failure (14.0%) occurred. The Kaplan-Meier survival curves illustrating the occurrence of heart failure based on the presence of VV are shown in Fig 2. Participants with VV had an increased risk of heart failure. In the multivariate analysis, the VV group consistently showed an increased incidence risk of heart failure compared to the non-VV group, both before (HR, 1.174; 95% CI, 1.089–1.265; *P* < 0.001) and after PSM (HR, 1.171; 95% CI, 1.070–1.283; *P* < 0.001) (Table 2 and S1 Table).

**Table 2:** Results of Cox regression analysis for the association of varicose veins with incidence risk of heart failure.

Variables	Before PSM n = 390,436			After PSM 1:5 n = 30,024		
	Incidence rate (per 100,000 person-years)	Crude HR (95% CI)	Adjusted HR (95% CI)	Incidence rate (per 100,000 person-years)	Crude HR (95% CI)	Adjusted HR (95% CI)
Without varicose veins	1,173.161	ref	ref	1,240.589	ref	ref
With varicose veins		1.245 (1.155–1.342)	1.174 (1.089–1.265)		1.172 (1.077–1.276)	1.171 (1.070–1.283)

Abbreviations: PSM, propensity score matching; HR, hazard ratio; CI, confidence interval. Values from multivariate Cox regression models adjusted for age, sex, body mass index, household income, smoking status, alcohol consumption, regular physical activity, comorbidities and Charlson comorbidity index.

**Fig 3:**
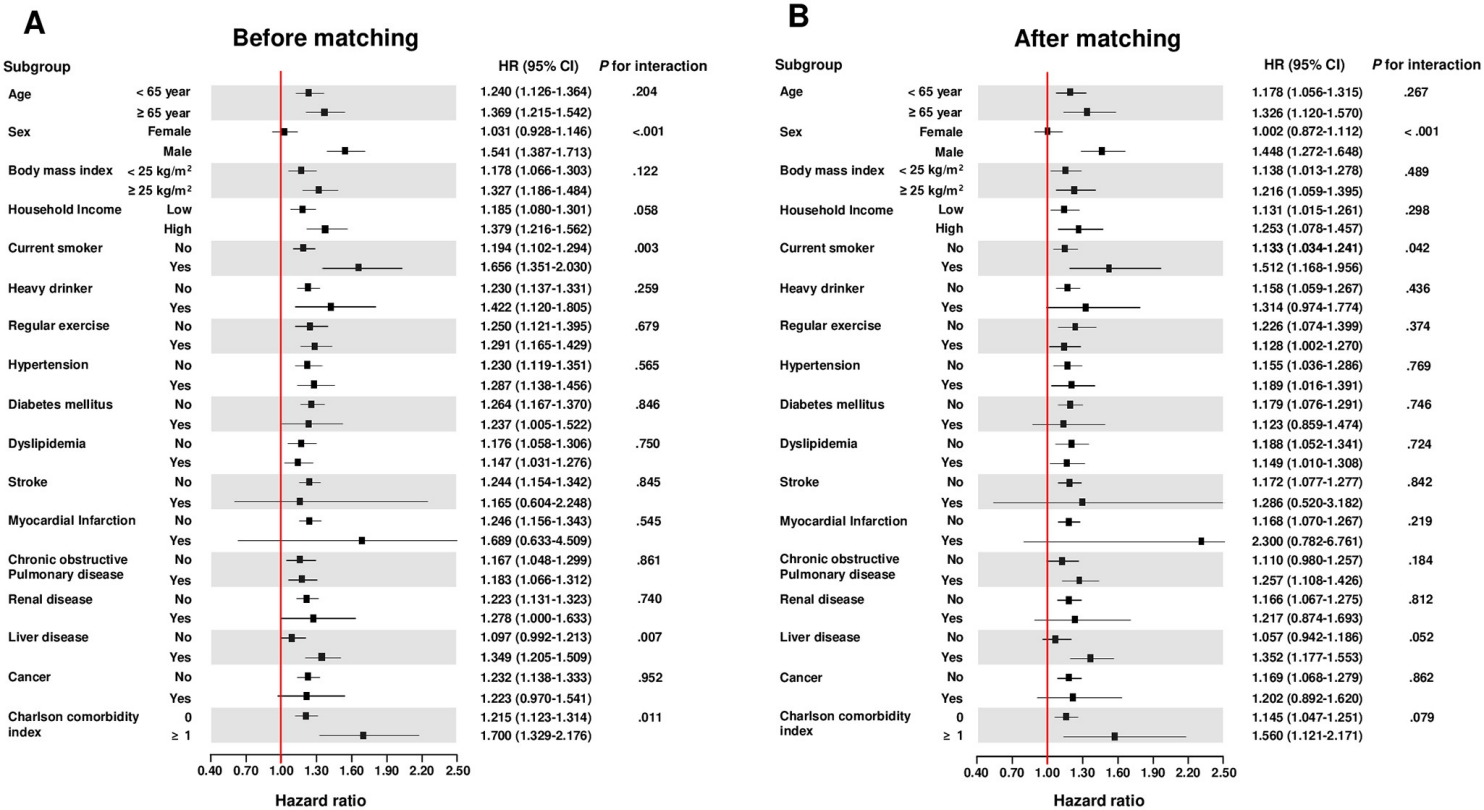
Forest plots showing the risk heart failure associated with the presence of varicose veins across various clinical subgroups.

In subgroup analysis, after PSM, the association between the presence of VV and the incidence risk of heart failure was consistently observed regardless of covariates. In addition, men and current smokers were found to have a significantly higher risk of heart failure compared to women and non-current smokers, respectively (Fig 3). In the landmark analysis, the relationship between the presence of VV and the incidence risk of heart failure was consistently demonstrated both before (HR, 1.190; 95% CI, 1.103–1.285; *P* < 0.001) and after PSM (HR, 1.144; 95% CI, 1.044–1.254; *P* = 0.041) (S2 Table and S1 Fig). In addition, the numbers of heart failure and mortality events are provided in S3 Table. When accounting for mortality as a competing risk, the SHR for heart failure in participants with VV was consistently increased compared to the non-VV group, both before (SHR, 1.126; 95% CI, 1.110–1.338), and after PSM (SHR, 1.146; 95% CI, 1.034–1.270) (S4 and S5 Tables).

### Association of varicose veins treatment with heart failure risk

Detailed frequency data of treatments and procedures are provided in S6 Table. When considering the association between procedure or treatment of VV and the incidence risk of heart failure, there was no significant difference between the VV group and non-VV group before (HR, 0.976; 95% CI, 0.824–1.128; *P* = 0.529) and after PSM (HR, 0.974; 95% CI, 0.893–1.055; *P* = 0.252) (S7 Table).

## Discussion

The main finding of our study is the significantly higher risk of heart failure in individuals with VV compared to matched controls, even in landmark analysis. Subgroup analysis revealed a significant increase in the risk of heart failure observed exclusively in males and current smokers with VV.

Research on the relationship between VV and heart failure has been reported as very limited to date, with conflicting results observed. Some previous studies have failed to find an association between heart failure and VV [10, 26–28], while others have suggested an increased risk of heart failure in patients with VV [29, 30]. The most recent nationwide cohort study in Taiwan revealed an increased adjusted HR for heart failure in patients with VV compared with matched controls [30]. On the other hand, a single-center prospective cohort study in Germany, even after adjusting for age, sex, and cardiovascular risk factors, found that chronic venous insufficiency was independently associated with cardiovascular diseases, including congestive heart failure, although VV did not demonstrate statistical significance [10]. Our study results add meaningful information on the association between the presence of VV and the risk of heart failure in the general population over time.

In our study, participants within the VV group were divided into two groups based on whether they received treatment to assess differences in heart failure risk. However, no significant difference in the occurrence of heart failure was found between the VV treated and untreated groups. These results may suggest that the treatment of VV does not significantly reverse underlying pathological processes, or that the treated group may comprise patients with more severe disease, making it challenging to observe a reduction in heart failure risk among those treated for VV. Alternatively, the lack of a significant difference in heart failure risk may indicate that VV does not have a clear causal impact on the development of heart failure.

Several mechanisms have been proposed to explain the association between the presence of VV and heart failure. First, shared risk factors between these conditions could account for the relationship. Many of the risk factors for VV are also recognized as risks for heart disease. In the Framingham study involving 3,822 adults, females with VV were found to be more obese, less physically active, have higher systolic blood pressure, and be older at menopause. However, these differences became insignificant after adjusting for body mass index and systolic blood pressure [26]. Nonetheless, despite adjusting for conditions including hypertension and diabetes, significant correlations between VV and cardiovascular diseases, including heart failure, have been demonstrated in another previous study [31]. Our study also reveals a significant association between VV and heart failure, even after controlling for body measurements, lifestyle habits, and comorbidities. Second, a more direct association between VV and heart failure can be exemplified by secondary varices characterized by pulsating veins, which may present with or without edema or ulceration and can potentially result from right heart failure [32, 33]. Third, there is a potential for endothelial dysfunction and inflammatory reactions to act commonly in the pathophysiological mechanisms of vascular diseases [31]. Research has shown that inflammatory biomarkers may assist in assessing the severity and prognosis of heart failure, suggesting their interconnectedness in common pathways between VV and heart failure [34, 35]. Pathological cardiac remodeling occurs as a characteristic pathophysiological feature of heart failure, involving molecular and biochemical pathways that lead to cellular alterations such as fibroblast proliferation, extracellular matrix reorganization, and accumulation of proinflammatory mediators [36]. It is possible that various changes in connective tissue and the involvement of inflammatory biomarkers in VV may share common risk factors contributing to the buildup of interstitial collagen fibers in the progression of heart failure. Fourth, genetics may represent another factor that could elucidate the association between VV and heart failure. For example, notable genes such as FOXC2 and NOTCH3, identified in familial VV, are also implicated as shared factors in the pathogenesis of heart failure [37, 38]. Although these genetic mutations may not directly cause heart failure, they suggest a potential mechanistic role in the disease manifestation process.

The results of our study suggest potentially important clinical implications. VV have traditionally been considered a peripheral venous disorder with limited systemic implications. However, our findings suggest a potential link between VV and heart failure, indicating that the relationship between these two conditions may be more complex than previously understood. While our study does not establish a direct causal relationship, the possibility of an interaction between venous insufficiency and cardiac function warrants further investigation to better understand how VV might influence the development or progression of heart failure. Given the potential for overlapping mechanisms, such as inflammation or endothelial dysfunction, it may be important to consider not only the treatment of the enlarged vessels associated with VV but also the management of comorbid conditions that could exacerbate cardiovascular issues. Future studies should focus on clarifying the nature of the link between VV and heart failure and exploring whether comprehensive management strategies addressing both venous and cardiac health could improve outcomes for patients with VV.

Our study has several limitations. First, the analysis did not include categorization based on disease severity, which prevented us from ascertaining the trend of risk according to severity. The health insurance claims data used in our study, which was based on ICD-10 diagnostic codes specifying the presence of complications such as inflammation or ulceration, does not correspond with the CEAP classification. Since the specified ICD-10 codes with complications were not validated, we chose to include the entire population of VV, whether with or without complications, which may encompass C2–6 according to the CEAP classification. In addition, the claims data used in this study do not effectively distinguish between left ventricular and right ventricular heart failure. Since the pathophysiological roles can differ significantly depending on which side of the heart is affected, this limitation prevents us from exploring the potential differential impacts of heart failure based on its location within the heart. Second, various prevalence rates of VV have been reported according to regions and ethnicities, ranging from < 1% to 73% in females and from 2% to 56% in males [7]. However, in our study, VV were diagnosed in relatively fewer individuals, with a prevalence of 0.88%. There is a possibility of selection bias due to VV not being documented in the claims data. Third, the possibility of an ethnic bias in our results could limit the wider applicability of our conclusions to different demographic groups. Therefore, it is crucial to carry out additional research encompassing diverse racial and ethnic populations. Fourth, apart from the adjusted covariates in our study, unmeasured confounders could potentially influence the outcomes. Lastly, the retrospective and observational nature of the study inherently limits our ability to establish cause-and-effect relationships. Establishing causality would require more rigorous experimental designs to account for potential confounding factors and to clearly define the direction of the relationship between VV and heart failure. Future studies will be necessary to clarify these causal relationships.

## Conclusions

This study longitudinally tracked NHIS claims data from the general Korean population and revealed a significant increase in the risk of heart failure among patients with VV compared to the control group. The presence of VV is likely associated with an increased risk of heart failure in the general population, suggesting that the risk of future heart failure should be considered when VV are present. Further research is needed to confirm the consistency of this relationship and to explore the pathophysiological mechanisms underlying the association between VV and heart failure.

## Supporting information

S1 Appendix Supplementary methods. (DOCX)

S1 Fig Kaplan-Meier survival curves illustrating the association between heart failure outcome and the presence of varicose veins, landmark analysis using a start time of 1 year after the index date. (TIF)

S1 Table Results of Cox regression analysis for the association of varicose veins with incidence risk of heart failure. (DOCX)

S2 Table Results of Cox regression analysis for the association of varicose veins with incidence risk of heart failure: A 1-year landmark analysis. (DOCX)

S3 Table Incidence counts of heart failure and mortality events. (DOCX)

S4 Table Results of Fine and Gray competing risk regression analysis for the association of varicose veins with incidence risk of heart failure. (DOCX)

S5 Table Results of Fine and Gray competing risk regression analysis for the association of varicose veins with incidence risk of heart failure. (DOCX)

S6 Table Frequency table of procedure code for population who received procedure/treatment for varicose veins. (DOCX)

S7 Table Results of Cox regression analysis for the association of procedure/treatment for varicose veins with incidence risk of heart failure. (DOCX)
