# Supplementary figures and images for: Association of varicose veins with the risk of heart failure: A nationwide cohort study
Source: PLoS One. 2025 Jan 7;20(1):e0316942. doi: 10.1371/journal.pone.0316942 (PMC11706482; doi:10.1371/journal.pone.0316942)

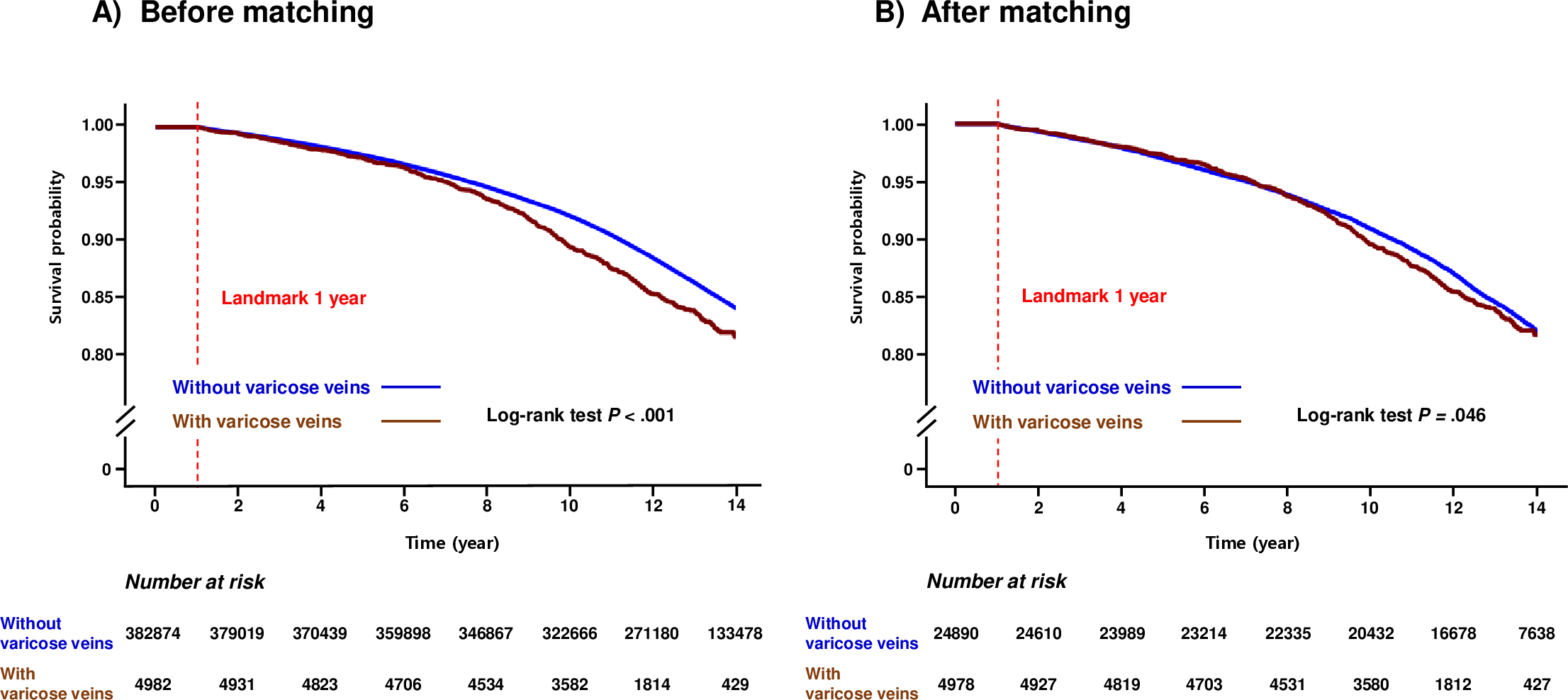

Supplement: S1 Fig — (TIF) [file pone.0316942.s002.tif]
