# Supplementary material for: Association of varicose veins with the risk of heart failure: A nationwide cohort study
Source: PLoS One. 2025 Jan 7;20(1):e0316942. doi: 10.1371/journal.pone.0316942 (PMC11706482; doi:10.1371/journal.pone.0316942)
Supplement: S1 Table — (DOCX) [file pone.0316942.s003.docx]

**S1 Table.** Results of Cox regression analysis for the association of varicose veins with incidence risk of heart failure.

| Variables | Before PSM  n = 390,436 | After PSM 1:5 n = 30,024 |
| --- | --- | --- |
|  | Adjusted HR  (95% CI) | Adjusted HR  (95% CI) |
| Without varicose veins | ref | ref |
| With varicose veins | 1.174 (1.089–1.265) | 1.171 (1.070–1.283) |
| Age, years | 1.068 (1.067–1.069) | 1.067 (1.063–1.072) |
| Sex |  |  |
| Male | ref | ref |
| Female | 0.922 (0.903–0.941) | 0.924 (0.842–1.013) |
| Body mass index (kg/m^2^) | 1.039 (1.036–1.042) | 1.045 (1.032–1.058) |
| Household income |  |  |
| T1 | ref | ref |
| T2 | 0.994 (0.974–1.014) | 1.001 (0.915–1.096) |
| T3 | 0.899 (0.880–0.919) | 0.909 (0.828–0.998) |
| Smoking status |  |  |
| Never | ref | ref |
| Former | 1.093 (1.059–1.129) | 1.078 (0.940–1.238) |
| Current | 1.286 (1.254–1.319) | 1.299 (1.137–1.483) |
| Alcohol consumption (days/week) |  |  |
| None | ref | ref |
| 1–2 times | 0.896 (0.876–0.917) | 0.860 (0.778–0.950) |
| 3–4 times | 0.902 (0.868–0.937) | 0.832 (0.701–0.987) |
| ≥ 5 times | 0.979 (0.938–1.021) | 0.899 (0.727–1.111) |
| Regular physical activity (days/week) |  |  |
| None | ref | ref |
| 1–4 days | 0.875 (0.858–0.892) | 0.925 (0.850–1.007) |
| ≥ 5 days | 0.885 (0.863–0.909) | 0.901 (0.813–0.998) |
| Comorbidities |  |  |
| Hypertension | 1.277 (1.254–1.301) | 1.316 (1.212–1.428) |
| Diabetes mellitus | 1.178 (1.150–1.207) | 1.228 (1.090–1.384) |
| Dyslipidemia | 1.248 (1.224–1.272) | 1.189 (1.096–1.291) |
| Stroke | 1.460 (1.349–1.581) | 1.409 (0.997–1.992) |
| Myocardial Infarction | 3.098 (2.801–3.427) | 2.976 (1.582–5.600) |
| COPD | 1.272 (1.250–1.295) | 1.201 (1.114–1.295) |
| Renal disease | 1.219 (1.176–1.263) | 1.280 (1.110–1.476) |
| Liver disease | 1.132 (1.109–1.155) | 1.186 (1.092–1.288) |
| Cancer | 1.173 (1.136–1.211) | 1.256 (1.104–1.429) |
| Charlson comorbidity index |  |  |
| 0 | ref | ref |
| 1 | 1.075 (1.041–1.111) | 1.183 (1.022–1.370) |
| 2 or more | 1.149 (1.047–1.260) | 1.361 (0.849–2.181) |

Abbreviations: PSM, propensity score matching; HR, hazard ratio; CI, confidence interval; T, tertiles; COPD, chronic obstructive pulmonary disease.
