# Supplementary material for: Association of varicose veins with the risk of heart failure: A nationwide cohort study
Source: PLoS One. 2025 Jan 7;20(1):e0316942. doi: 10.1371/journal.pone.0316942 (PMC11706482; doi:10.1371/journal.pone.0316942)
Supplement: S2 Table — (DOCX) [file pone.0316942.s004.docx]

**S2 Table.** Results of Cox regression analysis for the association of varicose veins with incidence risk of heart failure: A 1-year landmark analysis.

| Variables | Before PSM  n = 387,856 | | | After PSM 1:5 n = 29,868 | | |
| --- | --- | --- | --- | --- | --- | --- |
|  | Incidence rate  (per 100,000 person-years) | Crude HR  (95% CI) | Adjusted HR  (95% CI) | Incidence rate  (per 100,000 person-years) | Crude HR  (95% CI) | Adjusted HR  (95% CI) |
| Without varicose veins | 1,131.795 | ref | ref | 1,266.942 | ref | ref |
| With varicose veins |  | 1.259 (1.167–1.359) | 1.190 (1.103–1.285) |  | 1.118 (1.026–1.219) | 1.144 (1.044–1.254) |

Abbreviations: PSM, propensity score matching; HR, hazard ratio; CI, confidence interval. Values from multivariate Cox regression models adjusted for age, sex, body mass index, household income, smoking status, alcohol consumption, regular physical activity, comorbidities and Charlson comorbidity index.
