# Supplementary material for: Association of varicose veins with the risk of heart failure: A nationwide cohort study
Source: PLoS One. 2025 Jan 7;20(1):e0316942. doi: 10.1371/journal.pone.0316942 (PMC11706482; doi:10.1371/journal.pone.0316942)
Supplement: S3 Table — (DOCX) [file pone.0316942.s005.docx]

**S3 Table**. Incidence counts of heart failure and mortality events

|  | Before PSM | | | After PSM 1:5 | | |
| --- | --- | --- | --- | --- | --- | --- |
|  | Total | Without varicose veins | With varicose veins | Total | Without varicose veins | With varicose veins |
|  | n (%) | n (%) | n (%) | n (%) | n (%) | n (%) |
| No event | 324,506 (82.19) | 320,128 (82.13) | 4,378 (86.27) | 25,238 (82.95) | 20,862 (82.28) | 4,376 (86.29) |
| Heart failure | 35,654 (9.03) | 35,207 (9.03) | 447 (8.81) | 2,864 (8.78) | 2,419 (9.54) | 445 (8.78) |
| All-cause mortality | 34,683 (8.78) | 34,433 (8.83) | 250 (4.93) | 2,324 (4.93) | 2,074 (8.18) | 250 (4.93) |
