# Supplementary material for: Association of varicose veins with the risk of heart failure: A nationwide cohort study
Source: PLoS One. 2025 Jan 7;20(1):e0316942. doi: 10.1371/journal.pone.0316942 (PMC11706482; doi:10.1371/journal.pone.0316942)
Supplement: S4 Table — (DOCX) [file pone.0316942.s006.docx]

**S4 Table**. Results of Fine and Gray competing risk regression analysis for the association of varicose veins with incidence risk of heart failure

| Variables | Before PSM  n = 394,843 | | | After PSM 1:5 n = 30,426 | | |
| --- | --- | --- | --- | --- | --- | --- |
|  | Incidence rate  (per 100,000 person - years) | Crude SHR  (95% CI) | Adjusted SHR  (95% CI) | Incidence rate  (per 100,000 person - years) | Crude SHR  (95% CI) | Adjusted SHR  (95% CI) |
| Without varicose veins | 731.069 | ref | ref | 800.289 | ref | ref |
| With varicose veins |  | 1.218 (1.110 - 1.338) | 1.126 (1.024 - 1.237) |  | 1.126 (1.017 - 1.246) | 1.146 (1.034 - 1.270) |

Abbreviations: PSM, propensity score matching; n, number; SHR, subhazard ratio; CI, confidence interval. Values from multivariate Cox regression models adjusted for age, sex, body mass index, household income, smoking status, alcohol consumption, regular physical activity, comorbidities, and Charlson comorbidity index.
