# Supplementary material for: Association of varicose veins with the risk of heart failure: A nationwide cohort study
Source: PLoS One. 2025 Jan 7;20(1):e0316942. doi: 10.1371/journal.pone.0316942 (PMC11706482; doi:10.1371/journal.pone.0316942)
Supplement: S5 Table — (DOCX) [file pone.0316942.s007.docx]

**S5 Table**. Results of Fine and Gray competing risk regression analysis for the association of varicose veins with incidence risk of heart failure.

| Variables | Before PSM n = 394,843 | After PSM 1:5 n = 30,426 |
| --- | --- | --- |
|  | Adjusted SHR  (95% CI) | Adjusted SHR  (95% CI) |
| Without varicose veins | ref | ref |
| With varicose veins | 1.126 (1.024 - 1.237) | 1.146 (1.034 - 1.270) |
| Age, years | 1.062 (1.061 - 1.064) | 1.063 (1.059 - 1.067) |
| Sex |  |  |
| Male | ref | Ref |
| Female | 0.960 (0.936 - 0.986) | 0.866 (0.790 - 0.949) |
| Body mass index (kg/m2) | 1.061 (1.057 - 1.064) | 1.057 (1.044 - 1.071) |
| Household income |  |  |
| Low | ref | ref |
| Middle | 0.990 (0.965 - 1.016) | 1.020 (0.930 - 1.118) |
| High | 0.900 (0.876 - 0.925) | 0.920 (0.836 - 1.012) |
| Smoking status |  |  |
| Never | ref | ref |
| Former | 1.082 (1.039 - 1.127) | 0.990 (0.862 - 1.137) |
| Current | 1.225 (1.186 - 1.266) | 1.095 (0.951 - 1.260) |
| Alcohol consumption (days/week) |  |  |
| None | ref | ref |
| 1 - 2 times | 0.909 (0.883 - 0.936) | 0.921 (0.830 - 1.021) |
| 3 - 4 times | 0.862 (0.820 - 0.906) | 0.847 (0.705 - 1.018) |
| ≥ 5 times | 0.903 (0.855 - 0.953) | 0.943 (0.764 - 1.164) |
| Regular physical activity (days/week) |  |  |
| None | ref | ref |
| 1 - 4 days | 0.897 (0.875 - 0.919) | 0.936 (0.858 - 1.021) |
| ≥ 5 days | 0.889 (0.860 - 0.918) | 0.890 (0.806 - 0.983) |
| Comorbidities |  |  |
| Hypertension | 1.350 (1.319 - 1.382) | 1.293 (1.191 - 1.405) |
| Diabetes mellitus | 1.125 (1.092 - 1.159) | 1.183 (1.057 - 1.324) |
| Dyslipidemia | 1.340 (1.307 - 1.374) | 1.303 (1.195 - 1.420) |
| Stroke | 1.116 (1.006 - 1.237) | 1.188 (0.856 - 1.649) |
| Myocardial Infarction | 3.195 (2.833 - 3.604) | 3.613 (2.270 - 5.751) |
| COPD | 1.276 (1.248 - 1.305) | 1.284 (1.189 - 1.386) |
| Renal disease | 1.244 (1.191 - 1.299) | 1.260 (1.101 - 1.442) |
| Liver disease | 1.084 (1.057 - 1.112) | 1.102 (1.013 - 1.199) |
| Cancer | 1.058 (1.016 - 1.102) | 1.083 (0.955 - 1.228) |
| Charlson comorbidity index |  |  |
| 0 | ref | ref |
| 1 | 1.061 (1.018 - 1.106) | 1.152 (0.996 - 1.332) |
| ≥ 2 | 1.126 (1.004 - 1.263) | 1.343 (0.912 - 1.978) |

Abbreviations: PSM, propensity score matching; n, number; SHR, subhazard ratio; CI, confidence interval. COPD, chronic obstructive pulmonary disease.
