# Supplementary material for: Association of varicose veins with the risk of heart failure: A nationwide cohort study
Source: PLoS One. 2025 Jan 7;20(1):e0316942. doi: 10.1371/journal.pone.0316942 (PMC11706482; doi:10.1371/journal.pone.0316942)
Supplement: S6 Table — (DOCX) [file pone.0316942.s008.docx]

**S6 Table.** Frequency table of procedure code for population who received procedure/treatment for varicose veins.

| Varicose veins treatment group, n = 1,959 | | | | | | | | | | | |
| --- | --- | --- | --- | --- | --- | --- | --- | --- | --- | --- | --- |
| Number of treatment = 1 | | Number of treatment = 2 | | Number of treatment = 3 | | Number of treatment = 4 | | Number of treatment = 5 | | Number of treatment = 6 | |
| Procedure code | n (%) | Procedure code | n (%) | Procedure code | n (%) | Procedure code | n (%) | Procedure code | n (%) | Procedure code | n (%) |
| O0261 | 115 (5.8) | O0215 & O0216 | 27 (1.4) | O0215 & O0216 & O0217 | 6 (0.3) | O0261 & O0263 & O0264 & O0265 | 1 (0.1) | O0263 & O0264 & O0265 & O0266 & O2052 | 1 (0.1) | O0263 & O0265 & O0266 & O0267 & O0216 & O0217 | 1 (0.1) |
| O0262 | 67 (3.4) | O0215 & O0217 | 7 (0.4) | O0261 & O0215 & O0216 | 1 (0.1) | O0261 & O0263 & O0264 & O2052 | 1 (0.1) |  |  |  |  |
| O0263 | 129 (6.5) | O0216 & O0217 | 25 (1.3) | O0261 & O0215 & O0217 | 1 (0.1) | O0261 & O0263 & O0265 & O0216 | 2 (0.1) |  |  |  |  |
| O0264 | 160 (7.1) | O0261 & O0215 | 9 (0.5) | O0261 & O0263 & O2052 | 1 (0.1) | O0261 & O0264 & O0265 & O2052 | 1 (0.1) |  |  |  |  |
| O0265 | 238 (12.1) | O0261 & O0216 | 4 (0.2) | O0261 & O0265 & O2052 | 1 (0.1) | O0261 & O0265 & O0215 & O0217 | 1 (0.1) |  |  |  |  |
| O0266 | 147 (7.5) | O0261 & O0262 | 4 (0.2) | O0261 & O2052 & O0215 | 2 (0.1) | O0262 & O0264 & O0216 & O0217 | 1 (0.1) |  |  |  |  |
| O0267 | 11 (0.6) | O0261 & O0263 | 7 (0.4) | O0262 & O0215 & O0216 | 1 (0.1) | O0262 & O0264 & O0266 & O2052 | 1 (0.1) |  |  |  |  |
| O2052 | 272 (12.9) | O0261 & O0265 | 3 (0.2) | O0262 & O0215 & O0217 | 1 (0.1) | O0262 & O2052 & O0215 & O0216 | 1 (0.1) |  |  |  |  |
| O0215 | 205 (9.4) | O0261 & O0266 | 1 (0.1) | O0262 & O0264 & O0265 | 2 (0.1) | O0263 & O0264 & O0265 & O2052 | 1 (0.1) |  |  |  |  |
| O0216 | 59 (3.0) | O0261 & O0267 | 1 (0.1) | O0262 & O0264 & O0266 | 1 (0.1) | O0263 & O0265 & O0266 & O0217 | 1 (0.1) |  |  |  |  |
| O0217 | 61 (3.1) | O0261 & O2052 | 11 (0.6) | O0262 & O0266 & O0217 | 1 (0.1) | O0264 & O0265 & O2052 & O0217 | 1 (0.1) |  |  |  |  |
|  |  | O0262 & O0215 | 3 (0.2) | O0263 & O0215 & O0216 | 1 (0.1) | O0264 & O0266 & O2052 & O0215 | 1 (0.1) |  |  |  |  |
|  |  | O0262 & O0216 | 2 (0.1) | O0263 & O0215 & O0217 | 1 (0.1) | O0264 & O0266 & O2052 & O0216 | 1 (0.1) |  |  |  |  |
|  |  | O0262 & O0217 | 2 (0.1) | O0263 & O0264 & O0265 | 2 (0.1) | O0265 & O0215 & O0216 & O0217 | 1 (0.1) |  |  |  |  |
|  |  | O0262 & O0263 | 5 (0.3) | O0263 & O0265 & O0216 | 1 (0.1) |  |  |  |  |  |  |
|  |  | O0262 & O0264 | 4 (0.2) | O0263 & O0265 & O0266 | 1 (0.1) |  |  |  |  |  |  |
|  |  | O0262 & O0265 | 5 (0.3) | O0263 & O0266 & O0216 | 1 (0.1) |  |  |  |  |  |  |
|  |  | O0262 & O0266 | 2 (0.1) | O0263 & O0266 & O0217 | 3 (0.2) |  |  |  |  |  |  |
|  |  | O0262 & O2052 | 8 (0.4) | O0263 & O2052 & O0215 | 1 (0.1) |  |  |  |  |  |  |
|  |  | O0263 & O0215 | 3 (0.2) | O0264 & O0215 & O0216 | 3 (0.2) |  |  |  |  |  |  |
|  |  | O0263 & O0216 | 1 (0.1) | O0264 & O0216 & O0217 | 1 (0.1) |  |  |  |  |  |  |
|  |  | O0263 & O0217 | 9 (0.5) | O0264 & O0265 & O0215 | 2 (0.1) |  |  |  |  |  |  |
|  |  | O0263 & O0264 | 14 (0.7) | O0264 & O0265 & O0216 | 2 (0.1) |  |  |  |  |  |  |
|  |  | O0263 & O0265 | 13 (0.7) | O0264 & O0265 & O0266 | 2 (0.1) |  |  |  |  |  |  |
|  |  | O0263 & O0266 | 3 (0.2) | O0264 & O2052 & O0215 | 2 (0.1) |  |  |  |  |  |  |
|  |  | O0263 & O0267 | 1 (0.1) | O0264 & O2052 & O0217 | 1 (0.1) |  |  |  |  |  |  |
|  |  | O0263 & O2052 | 12 (0.6) | O0265 & O0215 & O0216 | 3 (0.2) |  |  |  |  |  |  |
|  |  | O0264 & O0215 | 10 (0.5) | O0265 & O0215 & O0217 | 1 (0.1) |  |  |  |  |  |  |
|  |  | O0264 & O0216 | 1 (0.1) | O0265 & O0216 & O0217 | 1 (0.1) |  |  |  |  |  |  |
|  |  | O0264 & O0217 | 11 (0.6) | O0265 & O0266 & O0215 | 1 (0.1) |  |  |  |  |  |  |
|  |  | O0264 & O0265 | 13 (0.7) | O0265 & O0266 & O0216 | 2 (0.1) |  |  |  |  |  |  |
|  |  | O0264 & O0266 | 26 (1.3) | O0265 & O0266 & O0217 | 3 (0.2) |  |  |  |  |  |  |
|  |  | O0264 & O2052 | 15 (0.8) | O0265 & O0266 & O2052 | 3 (0.2) |  |  |  |  |  |  |
|  |  | O0265 & O0215 | 10 (0.5) | O0265 & O0267 & O2052 | 1 (0.1) |  |  |  |  |  |  |
|  |  | O0265 & O0216 | 7 (0.4) | O0265 & O2052 & O0215 | 2 (0.1) |  |  |  |  |  |  |
|  |  | O0265 & O0217 | 10 (0.5) | O0265 & O2052 & O0216 | 1 (0.1) |  |  |  |  |  |  |
|  |  | O0265 & O0266 | 18 (0.9) | O0265 & O2052 & O0217 | 1 (0.1) |  |  |  |  |  |  |
|  |  | O0265 & O0267 | 1 (0.1) | O0266 & O0215 & O0216 | 1 (0.1) |  |  |  |  |  |  |
|  |  | O0265 & O2052 | 15 (0.8) | O0266 & O0215 & O0217 | 1 (0.1) |  |  |  |  |  |  |
|  |  | O0266 & O0215 | 18 (0.9) | O0266 & O0216 & O0217 | 1 (0.1) |  |  |  |  |  |  |
|  |  | O0266 & O0216 | 1 (0.1) | O0266 & O2052 & O0217 | 1 (0.1) |  |  |  |  |  |  |
|  |  | O0266 & O0217 | 18 (0.9) | O2052 & O0215 & O0216 | 3 (0.2) |  |  |  |  |  |  |
|  |  | O0266 & O2052 | 9 (0.5) | O2052 & O0216 & O0217 | 3 (0.2) |  |  |  |  |  |  |
|  |  | O0267 & O0215 | 1 (0.1) |  |  |  |  |  |  |  |  |
|  |  | O2052 & O0215 | 13 (0.7) |  |  |  |  |  |  |  |  |
|  |  | O2052 & O0216 | 12 (0.6) |  |  |  |  |  |  |  |  |
|  |  | O2052 & O0217 | 12 (0.6) |  |  |  |  |  |  |  |  |

The procedure code names of the treatment corresponding to each codes are as follows: Saphenous vein ligation & stab avulsion + perforator ligation(O0261); Saphenous vein ligation & stab avulsion – perforator ligation (O0262); Segmental stripping & stab avulsion + perforator ligation(O0263); Segmental stripping & stab avulsion – perforator ligation(O0264); Total stripping & stab avulsion + perforator ligation(O0265); Total stripping & stab avulsion – perforator ligation(O0266); Varicose vein operation, others(perineum)(O0267); Local resection(O2052); 1–3 sites(O0215); 4–6 sites (O0216); More than 7 sites(O0217).
