# Supplementary material for: Association of varicose veins with the risk of heart failure: A nationwide cohort study
Source: PLoS One. 2025 Jan 7;20(1):e0316942. doi: 10.1371/journal.pone.0316942 (PMC11706482; doi:10.1371/journal.pone.0316942)
Supplement: S7 Table — (DOCX) [file pone.0316942.s009.docx]

**S7 Table.** Results of Cox regression analysis for the association of procedure/treatment for varicose veins with incidence risk of heart failure.

| Variables | Before PSM  n = 5,008 | After PSM 1:1 n = 3,834 |
| --- | --- | --- |
|  | Adjusted HR  (95% CI) | Adjusted HR  (95% CI) |
| Without treatment | ref | ref |
| With treatment | 0.976 (0.824–1.128) | 0.974 (0.893–1.055) |
| Age, years | 1.076 (1.066–1.086) | 1.074 (1.062–1.087) |
| Sex |  |  |
| Male | ref | ref |
| Female | 0.863 (0.715–1.042) | 0.836 (0.669–1.044) |
| Body mass index (kg/m^2^) | 1.051 (1.024–1.080) | 1.044 (1.010–1.079) |
| Household income |  |  |
| T1 | ref | ref |
| T2 | 1.000 (0.828–1.208) | 1.001(0.805–1.246) |
| T3 | 0.975 (0.804–1.182) | 0.878(0.696–1.107) |
| Smoking status |  |  |
| Never | ref | ref |
| Former | 1.190(0.913–1.552) | 1.231 (0.906–1.674) |
| Current | 1.537(1.193–1.980) | 1.330 (0.967–1.831) |
| Alcohol consumption (days/week) |  |  |
| None | ref | ref |
| 1–2 times | 0.926 (0.757–1.133) | 0.970 (0.772–1.220) |
| 3–4 times | 0.970 (0.693–1.357) | 0.954 (0.625–1.456) |
| ≥ 5 times | 0.754 (0.486–1.171) | 0.477 (0.250–0.911) |
| Regular physical activity (days/week) |  |  |
| None | ref | ref |
| 1–4 days | 0.861 (0.723–1.024) | 0.802 (0.652–0.988) |
| ≥ 5 days | 0.763 (0.620–0.939) | 0.697 (0.544–0.894) |
| Comorbidities |  |  |
| Hypertension | 1.326 (1.122–1.566) | 1.295 (1.059–1.585) |
| Diabetes mellitus | 1.055 (0.835–1.333) | 0.927 (0.685–1.255) |
| Dyslipidemia | 1.075 (0.906–1.277) | 1.075 (0.875–1.320) |
| Stroke | 1.413 (0.727–2.746) | 1.480 (0.653–3.350) |
| Myocardial Infarction | 3.075 (1.121–8.440) | 12.049 (2.920–49.713) |
| COPD | 1.295 (1.109–1.512) | 1.372 (1.140–1.652) |
| Renal disease | 1.175 (0.896–1.540) | 1.088 (0.762–1.554) |
| Liver disease | 1.383 (1.172–1.632) | 1.548 (1.271–1.885) |
| Cancer | 1.267 (0.984–1.630) | 1.177 (0.855–1.619) |
| Charlson comorbidity index |  |  |
| 0 | ref | ref |
| 1 | 1.156 (0.865–1.546) | 1.200 (0.834–1.725) |
| 2 or more | 1.975 (1.062–3.673) | 0.892 (0.324–2.456) |

Abbreviations: PSM, propensity score matching; HR, hazard ratio; CI, confidence interval; T, tertiles; COPD, chronic obstructive pulmonary disease.
